# Specificity of Sensory and Motor Neurons Associated with BL40 and GB30 in the Rat: A Dual Fluorescent Labeling Study

**DOI:** 10.1155/2013/643403

**Published:** 2013-08-21

**Authors:** Jingjing Cui, Lijuan Ha, Xinlong Zhu, Fuchun Wang, Xianghong Jing, Wanzhu Bai

**Affiliations:** ^1^Institute of Acupuncture and Moxibustion, China Academy of Chinese Medical Sciences, Beijing 100700, China; ^2^Changchun University of Chinese Medicine, Changchun 130117, China

## Abstract

The purpose of this study is to investigate the specific innervations on “Weizhong” (BL40) and “Huantiao” (GB30) by using a dual neural tracing technique. After Alexa Fluor 488 and 594 conjugates of cholera toxin subunit B (AF488/594-CTB) were, respectively, injected into BL40 and GB30 in the same rat, the labeled sensory and motor neurons were examined in the rat's dorsal root ganglia (DRGs) and spinal cord at thoracic (T) and lumbar (L) segments with a laser scanning confocal microscope. In the cases of BL40 injection, AF488-CTB labeled sensory and motor neurons were located in L_2–6_ DRGs and on the mediolateral part of spinal ventral horn from L_3_ to L_5_ segments, respectively. By contrast, in the cases of GB30 injection, AF594-CTB labeled sensory and motor neurons were distributed in T_13_-L_6_ DRGs and on the anterolateral part of spinal ventral horn from L_1_ to L_5_ segments, respectively. These results indicate that the sensory and motor neurons associated with BL40 and GB30 are located in different spinal segments and regions in the nervous system, providing the neuroanatomical evidence to serve the specificity of acupoints.

## 1. Introduction

Both “Weizhong” (BL40) and “Huantiao” (GB30) are commonly used for acupuncture treatment on lumbar and lower limb disorders [1–3], which could be traced back to “A Verse on the Twelve Heaven-Star Points” compiled by Ma Danyang in Song dynasty, showing its important historical position in acupuncture treatment. Although BL40 and GB30 play active roles in treating common ailments, such as sciatica, lumbar intervertebral disc herniation, and muscle strain [1–3], the underlying mechanism for their effectiveness remains unclear. Recent studies suggest that the effect of acupuncture is highly correlated with the activation of the nervous system [4–8]. Thus, to understand the neural properties of different acupoints may play a pivotal role in exploring the mechanism of acupuncture. In line with this kind of studies, how to apply an effective approach for further investigating the specific innervations on different acupoints is an important task to know the specificity of acupoints.

Since neural tracing technique was introduced into the acupuncture research, it has opened a new field to understand the neural properties of acupoints at the cellular level, especially the sensory and motor innervation on acupoints [9–11]. As a new generation of sensitive tracer [12, 13], Alexa Fluor 488 and 594 conjugates of cholera toxin subunit B (AF488/594-CTB) have been proven to be a potential approach in morphological study on the neural properties of acupoints [14, 15]. In order to reveal the characteristics of the neurons related to different acupoints, in this study, BL40 and GB30 were, respectively, labeled AF488/594-CTB in rats. By using this neural tracing technique, the distribution of the sensory and motor neurons associated with BL40 and GB30 could be revealed, which should benefit the understanding of the specificity of acupoints from the perspective of neuroanatomy.

## 2. Material and Methods

### 2.1. Subjects

Four adult male Sprague Dawley rats (6-7 weeks old, weight 225 ± 25 g) at clean level were used in the present study. Experimental animals were provided by the Institute of Laboratory Animal Sciences, Chinese Academy of Medical Sciences. The license number is SCKX (JUN) 2007-004. All animals were housed in a 12 h light/dark cycle with controlled temperature and humidity and allowed free access to food and water. The handling and care of experimental animals conformed to the regulations provided by the National Institutes of Health Guide for the Care and Use of Laboratory Animals [16].

### 2.2. Research Protocol

#### 2.2.1. Microinjection of AF488/594-CTB

**Figure 1:**
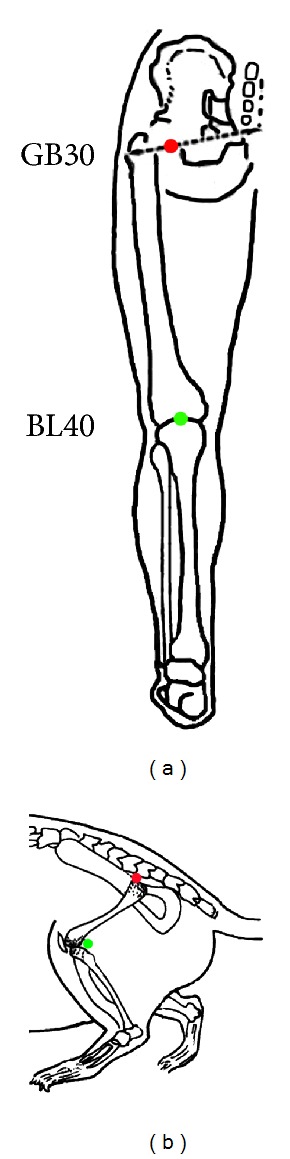
Illustration of the acupoints of Huantiao (GB30, red) and Weizhong (BL40, green) in human (a) and rat (b).

Injection was performed on both BL40 and GB30 in the same rat. Based on the classification of traditional Chinese medicine, BL40 belongs to the bladder meridian of foot Taiyang and GB30 is located on the gallbladder meridian of foot Shaoyang. The AF488/594-CTB (Invitrogen-Molecular Probes, Eugene, OR, USA) were used as tracers to, respectively, determine the distribution of neurons related to BL40 and GB30. According to the principle of comparative anatomy, BL40 is located at the midpoint of the popliteal crease, and GB30 is situated at the junction of the lateral 1/3 and medial 2/3 of the line connecting the prominence of greater trochanter of femur with the sacral hiatus on the rat, corresponding to BL40 and GB30 on the human body (Figure 1). Under anesthesia with isoflurane (Litian, Jiupai Pharmaceutical Co. Ltd, Hebei, China) controlled by small animal anesthesia machine (VMR, Matrx, Midmark, USA), 8 *μ*L of 0.1% AF488/594-CTB were, respectively, injected into BL40 and GB30 on the left side with 10 *μ*L Hamilton syringe. The depth of injection was 4-5 mm. In order to prevent leakage of solution, the needle was kept for 1 min after injection and then slowly pulled out. When the rats awoke from anesthesia, they were put back in their cages.

#### 2.2.2. Perfusion

Three days (72 h) after injection, the rats were deeply anaesthetized by ether and transcardially perfused with 150 mL of 0.9% saline immediately followed by 300 mL of 4% paraformaldehyde in 0.1 M phosphate buffered solution (PB, pH 7.4). After perfusion, dorsal root ganglia (DRGs) and spinal cord from thoracic (T) 10 to lumbar (L) 6 segments were dissected out and put into the same fixative solution about 2–4 h, then changed into 25% sucrose PB (0.1 M, pH 7.4) at 4°C, and allowed to sink. The tissue on injection site was also dissected out for observing the local diffusion of tracers. The level of spinal segments was determined cytoarchitecturally, referred as to *The Rat Brain in Stereotaxic Coordinates* [17].

#### 2.2.3. Section

Serial sagittal sections of DRGs and transverse sections of spinal cord were cut at a thickness of 40 *μ*m on a freezing microtome (Thermo, Microm International GmbH, Germany). All sections were collected in order in a six-hole Petri dish with in 0.1 M PB (pH 7.4) and then stored in the refrigerator at 4°C.

Before observation, the sections were mounted on the microscope slides and coverslipped with 50% glycerin to improve visualization of labeling.

#### 2.2.4. Observation

The tissue samples were observed and recorded with a laser scanning confocal microscope (FV1000, Olympus Co., Tokyo, Japan). Digital images were then processed with Adobe Photoshop CS2 (Adobe Systems, San Jose, CA, USA). 

#### 2.2.5. Statistical Analysis

Data was expressed as mean ± standard deviation and processed with the statistical software SPSS 16.0.

## 3. Results

**Figure 2:**
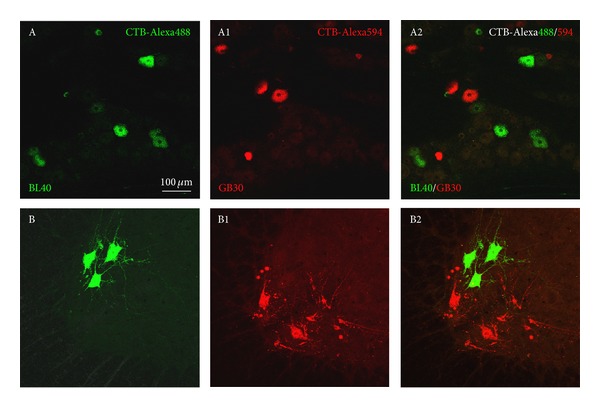
Representative sections from lumbar 4 dorsal root ganglion (DRG) and spinal cord showing the distribution of the labeled sensory neurons in DRG (A–A2) and motor neurons in spinal ventral horn (B–B2) after respective injection of AF488-CTB and AF594-CTB into BL40 and GB30 in the same rat. A, B: AF488-CTB labeled sensory (A) and motor (B) neurons related to BL40; A1, B1: AF594-CTB labeled sensory (A1) and motor (B1) neurons related to GB30; A2, B2: merged photo from A and A1(A2), B and B1(B2). Scale bar for all photos shown in A.

**Figure 3:**
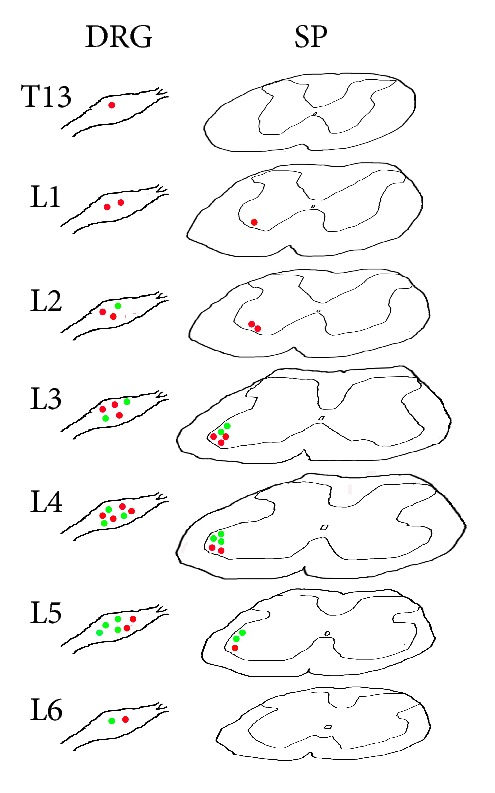
A series of line drawings through seven levels of dorsal root ganglia (DRGs) and approximately equal levels of spinal cord (SP) from thoracic (T) 13 to lumbar (L) 6 spinal segments showing the segmental and regional distribution of the sensory and motor neurons associated with BL40 (green) and GB30 (red), respectively.

The labeled sensory and motor neurons with AF488/594-CTB were located ipsilaterally on the injection side, in which AF488-CTB labeling was demonstrated in fluorescent green, and AF594-CTB was shown in fluorescent red (Figure 2). The neurons associated with BL40 and GB30 were distributed separately on the thoracic and lumbar DRGs and spinal cord. No neural labeling was observed above the level of spinal cord. The segmental and regional distribution of the sensory and motor neurons associated with BL40 and GB30 was summarized in Figure 3. 

### 3.1. Sensory Innervation

**Figure 4:**
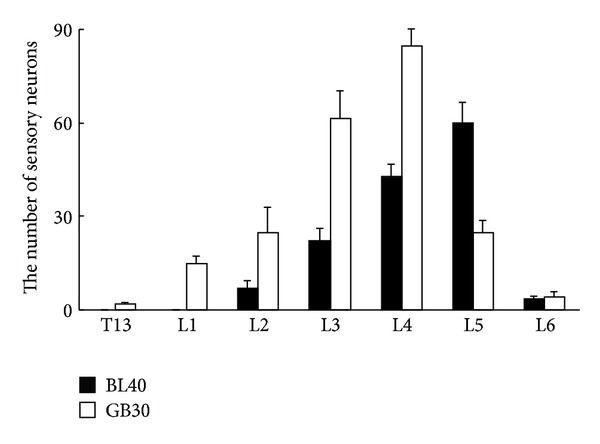
The average number of the AF488-CTB and AF594-CTB labeled sensory neurons in the DRGs ranging from thoracic (T) 13 to lumbar (L) 6 segments (mean ± standard deviation, *n* = 4), in which the sensory neurons associated with BL40 were distributed from L2 to L6 segments and those of GB30 distributed from T13 to L6.

In the cases of BL40 injection, the AF488-CTB labeled sensory neurons were detected in L_2_–L_6_ DRGs with high concentration in L_5_ DRGs (Figures 2(A) and 3). By contrast, in the cases of GB30 injection, the AF594-CTB labeled sensory neurons were detected in the T_13_–L_6_ DRGs with high concentration in L_4_ DRGs (Figures 2(A1) and 3). No double labeled sensory neurons were detected (Figure 2(A2)). In the four rats, a total of 538 AF488-CTB labeled sensory neurons (BL40) and 866 AF594-CTB labeled sensory neurons (GB30) were counted in the DRGs and arranged in order at different segments (Figure 4).

According to the size of soma diameter, the labeled sensory neurons were assigned to three classes: the large one (soma diameter > 50 *μ*m), the medium one (soma diameter between 30 *μ*m and 50 *μ*m), or the small one (soma diameter < 30 *μ*m) [18]. In both BL40 and GB30 injections, the small- and medium-sized sensory neurons each represented over 40% of the labeled neurons (Table 1). 

### 3.2. Motor Innervation

In the cases of BL40 injection, the AF488-CTB labeled motor neurons were distributed on the mediolateral part of spinal ventral horn from L_3_ to L_5_ segments and concentrated at L_4_ segment (Figures 2(B) and 3). By contrast, in the cases of GB30 injection, the AF594-CTB labeled motor neurons were distributed on the anterolateral part of spinal ventral horn from L_1_ to L_5_ segments with high concentration at L_3_ segment (Figures 2(B1) and 3). No double labeled motor neurons were observed (Figure 2(B2)). Because there is no distinct boundary among the different spinal segments, the figures were shown at approximate segment of spinal cord, and the labeled motor neurons were not counted separately. 

According to the size of soma diameter, the labeled motor neurons were divided into two classes: the large one (soma diameter > 25 *μ*m) or the small one (soma diameter ≤ 25 *μ*m), belonging to *α* and *γ* motor neurons, respectively [19]. The approximate 60 transverse sections from spinal cord were counted on every rat, resulting in a total of 136 AF488-CTB labeled motor neurons (BL40) and 174 AF594-CTB labeled motor neurons (GB30). The large- and small-sized motor neurons, respectively, represented 98.53% (134/136) and 1.47% (2/136) in the cases of BL40 injection and 92.53% (161/174) and 7.47% (13/174) in the cases of GB30 injection.

In addition, around the injection site, AF488-CTB and AF594-CTB were diffused in the muscles and subcutaneous tissues, but the scope of local diffusion was not over 1 mm from the center of injection site.

## 4. Discussion

By using a dual fluorescent labeling technique with AF488/594-CTB, we successfully revealed that the sensory and motor neurons associated with BL40 and GB30 were distributed separately in DRGs and spinal cord in rats, in which the segmental and regional arranged neurons closely correspond with individual acupoint, providing the neuroanatomical evidence to serve the specificity of acupoints. 

### 4.1. Technical Considerations

Recently, AF488/594-CTB are increasingly used for tracing the neural pathway in neuroscience research [12, 13]. Our recent studies also suggested that the technique of AF488/594-CTB is a proper choice to investigate the neural properties of different acupoints [14, 15]. Compared with tracers previously used in this field, such as propidium iodide (PI) and bisbenzimide (Bb) [20–22], the main advantages of AF488/594-CTB are their sensitivity and fluorescence persistence. Because the spectral range of specific fluorescent materials carried by AF488/594-CTB is concentrated and corresponds with the stimulating lights of a laser scanning confocal microscope, its neural labeling is more easily observed and identified without disturbance of nonspecific fluorescence labeling as observed with that of PI and Bb labeling [20–22]. Although it does not need complex staining processes to detect neural labeling of AF488/594-CTB like that of Horseradish Peroxidase (HRP) [11], it should be noted that AF488/594-CTB are only limited to the application of retrograde labeling the sensory and motor neurons in the tracing study and cannot be used for labeling transganglionic axonal terminals like that of HRP. Nevertheless, the present results provided sufficient evidence to propose that AF488/594-CTB are a suitable couple for dual fluorescent labeling in morphological research of acupuncture.

### 4.2. Distribution of Sensory and Motor Neurons

Previous topographic studies have shown that BL40 and GB30 are highly correlated with the tibial nerve and sciatic nerve, respectively, at the level of gross anatomy [23, 24]. However, at the cellular level, we still do not know the distribution of the sensory and motor neurons associated with both acupoints in the nervous system. In this study, we demonstrated that neurons related to BL40 and GB30 were distributed in a definite segmental and regional pattern, in which the sensory and motor neurons associated with GB30 distributed more extensively and higher than those of BL40 about one or two segments in DRGs and spinal ventral horn (Figure 3). Although with a part of segmental overlap, there were no dual labeled neurons in the present study. Thus it could be concluded that BL40 and GB30 received innervations from different sensory and motor neurons. In addition, the motor neurons related to BL40 and GB30 were distributed in the mediolateral and anterolateral regions of spinal ventral horn, respectively. This regional arrangement also supports the idea that the innervations on different acupoints originate from different neurons. 

It has been shown that peripheral areas on the body correlate orderly with the nervous system, which was named somatotopic organization [25–27]. Without exception, as a point of peripheral areas, every acupoint has its own corresponding neurons in the nervous system. Considering the distances from BL40 and GB30 to the truck of body, it is clear that the closer to the body trunk the acupoint is, the higher the neurons associated with the corresponding acupoint situate in the spinal segment. Similar results were also demonstrated in our previous observations on the acupoints of “Taixi” (KI3), “Chengshan” (BL 57), “Jinggu” (BL64), and “Dazhong” (KI4) [11, 14, 15]. Through these neural tracing studies on the different acupoints, we can speculate the innervations on acupoints according to their locations on the proximal, mid, and distal parts of hindlimb. Therefore, from the perspective of neuroanatomy, this study increases our understanding of the regular connections between the different acupoints and the nervous system.

### 4.3. The Subtype of Sensory and Motor Neurons

Large-, medium-, and small-sized sensory neurons were simultaneously labeled in both cases of BL40 and GB30 injections, which roughly correspond to A*α*/A*β*-, A*δ*-, and C-fibers, respectively [18, 28]. These three types of sensory neurons may play different roles in the processing of signals transmission due to their electrophysiological properties [29–31]. It was suggested that A*α*/A*β*-type fibers transmit messages of tactile sensation and proprioception, while A*δ*- and C-type fibers relay messages of nociception and thermal sensation [29–31]. Since different kinds of sensory neurons directly innervate the acupoints, it may be an important implication for further considering roles of these subtype neurons in the process of acupuncture stimulation. Besides the different kinds of sensory neurons, both *α* and *γ* motor neurons also participate in innervation on the BL40 and GB30. It should be another important consideration on the neural properties of different acupoints. Although we only provided the neuroanatomical evidence of BL40 and GB30 in this study, these results have served as a source of inspiration for investigating the specificity of acupoints at the cellular level.

### 4.4. The Significance for Clinical Practice

The relative specificity of acupoints is an important issue in the clinical treatment. To understand the neural properties of BL40 or GB30 should be of benefit for us to select proper one for acupuncture treatment according to the patient's symptoms. Given that the neurons associated with BL40 and GB30 concentrate on different spinal segments, we suggest that when the lumbar disorder occurred on L_4_, GB30 was recommended as main acupoint, while if it occurred on L_5_, BL40 was recommended. We can also simultaneously select both GB30 and BL40 to treat the ailment involving multiple spinal segments. Although this implication was supported by the perspective of neural pathway from the different acupoints to the nervous system, it remains to be verified in clinical practice.

## 5. Conclusion

In summary, we successfully demonstrated the specificity of distribution and subtype of the sensory and motor neurons associated with BL40 and GB30 in the rat by using a dual fluorescent labeling technique with AF488/594-CTB. These results suggest that regular connections between the different acupoints and the nervous system should be an important consideration during the acupuncture treatment.

## Figures and Tables

**Table 1:** Numbers and proportions of large-, medium-, and small-sized sensory neurons associated with BL40 and GB30 in the DRGs (number (%)).

Sensory neurons in the DRGs	BL40	GB30
Large-sized neuron	58 (10.78%)	149 (17.21%)
Medium-sized neuron	222 (41.26%)	357 (41.22%)
Small-sized neuron	258 (47.96%)	360 (41.57%)
